# Measurement of Trust in Automation: A Narrative Review and Reference Guide

**DOI:** 10.3389/fpsyg.2021.604977

**Published:** 2021-10-19

**Authors:** Spencer C. Kohn, Ewart J. de Visser, Eva Wiese, Yi-Ching Lee, Tyler H. Shaw

**Affiliations:** ^1^George Mason University, Fairfax, VA, United States; ^2^Warfighter Effectiveness Research Center, United States Air Force Academy, Colorado Springs, CO, United States

**Keywords:** trust, automation, autonomous, measures, measurement, self-report, behavioral, physiological

## Abstract

With the rise of automated and autonomous agents, research examining Trust in Automation (TiA) has attracted considerable attention over the last few decades. Trust is a rich and complex construct which has sparked a multitude of measures and approaches to study and understand it. This comprehensive narrative review addresses known methods that have been used to capture TiA. We examined measurements deployed in existing empirical works, categorized those measures into self-report, behavioral, and physiological indices, and examined them within the context of an existing model of trust. The resulting work provides a reference guide for researchers, providing a list of available TiA measurement methods along with the model-derived constructs that they capture including judgments of trustworthiness, trust attitudes, and trusting behaviors. The article concludes with recommendations on how to improve the current state of TiA measurement.

## Introduction

A critical factor of the success of human-machine teaming is the trust that the human teammate possesses in their machine teammate. Automation and autonomous systems offer greater consistency and accuracy than a human could provide, often in tasks that are too repetitive, fast, or dangerous for humans to perform. With these new teammates comes the opportunity for greater team performance – if the human operator trusts their autonomous teammate, the human may focus on their own tasks and strengths. The uncertainty and vulnerability that comes with trusting these machine partners fulfills the classic definition of trust as “…the attitude that an agent will help achieve an individual’s goals in a situation characterized by uncertainty and vulnerability” (Lee and See, 2004, p. 54). This definition parallels earlier definitions of trust in including risk and incentives at stake (Mayer et al., 1995). Given how crucial trust is to effective teaming and the increasing ubiquity of autonomous and automated teammates, interest in studying trust in those systems has correspondingly increased. These automated systems are the focus of applied research by their builders and basic research performed by academics who now have greater access to these tools.

The research approaches to the subject of trust are as varied as the products intended to inspire trust. In consequence, trust research is no longer the sole domain of a limited number of trust in automation (TiA) experts and is often performed by those who have direct access and an immediate interest in autonomous and automated systems. While the proliferation of this topic is a boon for the field, prior work has established that trust is too often measured by a small set of popular self-report measures (Kohn et al., 2020) or by popular measures that may be flawed (Gutzwiller et al., 2019). Novice researchers also have a troubling tendency to use custom measures that have not been previously validated (Kohn et al., 2020). Similarly, researchers may mis-conceptualize the constructs that they are capturing. TiA is a multi-faceted latent concept that defies easy conceptualization, yet trust measurement often purports to capture the simple construct “trust,” defying the complexity of this concept as established in existing trust models (e.g., Mayer et al., 1995; Lee and See, 2004; Hoff and Bashir, 2015). As a result, the selected trust measures used may be insensitive to the trust manipulation or may provide inconsistent or non-replicable results because they are capturing a facet of trust other than what the researchers intended.

Developing expertise in TiA research should not be necessary in order for researchers and practitioners to utilize trust measures. Rather, the field should provide reference tools that make selection and discussion of appropriate measures easier. Despite the many recent reviews and meta-analyses that have lent legitimacy to the topic of TiA and its sibling human-robot trust (See: Lee and See, 2004; Hancock et al., 2011; Hoff and Bashir, 2015; Schaefer, 2016; de Visser et al., 2020; Chiou and Lee, 2021), these reviews do not thoroughly review or discuss trust measures nor offer guidance about when or how different trust components may be captured. Researchers may turn to one of the new trust measurement methods that are introduced every year, but these works often only review the alternative measurement options in order to point out their deficiencies in comparison to the new method.

Therefore, we present a review work that serves as an educational tool, as well as a reference for those seeking to measure TiA – whether novices or experienced practitioners. This work will objectively examine and compare existing methods of measuring TiA, identify the facets of trust captured by each measure by contextualizing them within Mayer’s popular model of trust (1995), and describe the advantages, disadvantages, and usage of each category of trust measurement. A review of this magnitude has not been previously performed for trust measurement and is needed to improve the state of measurement in the field. This review intends to grant greater insight into what is being captured by this TiA measurements and make recommendations for improved accuracy and appropriateness of trust measurement.

### Focus of This Review

This review focuses on the measurement of TiA. TiA, as defined by Lee and See (2004, p. 54), will play an increasingly large role in our interactions with technology given the rapidly broadening capabilities of said technology. Automation is defined as “the execution by a machine agent (usually a computer) of a function that was previously carried out by a human” (Parasuraman and Riley, 1997, p. 231), while autonomy has been defined as systems that are “generative and learn, evolve and permanently change their functional capacities as a result of the input of operational and contextual information” (Hancock, 2017, p. 284). The primary differentiator between automation and autonomy is self-governance (Kaber, 2018). The current review focuses on the operator-automation dyad, a single operator supervising one or more automated agents (Parasuraman and Riley, 1997; Parasuraman, 2000). Examples include an operator using a bag-screening tool to detect hazards, a pilot supervising the auto-pilot of a plane, a driver interacting with the automated driving capabilities of a car, or an operator supervising multiple unmanned vehicles or interacting with a robot leader that in turn supervises other agents (e.g., Chen and Barnes, 2014). While the automation may consist of many component systems, this review focuses on operators collaborating with single instances of automation or automation that is viewed as a collective (See Geels-Blair et al., 2013, for a larger discussion of trusting automation as individual components versus a collective system). This interaction stands in contrast with the human-autonomy team, which focuses on teams of humans and autonomous agents which have distinct roles and interdependence in activities and outcomes (O’Neill et al., 2020). The operator-automation dyad and human-autonomy have been empirically found to be distinctly different (Walliser et al., 2019). With this contrast in mind, all discussion of trust measurement refers to the operator-automation dyad unless otherwise stated.

The automation may have a variety of characteristics: It may be solely expressed in software or may be embodied, such as a robot. It may possess a design that inspires anthropomorphism through facial features (DiSalvo et al., 2002; Phillips et al., 2018), labeling (Waytz et al., 2014), voice (Nass et al., 1997; Muralidharan et al., 2014), or etiquette (Parasuraman and Miller, 2004; de Visser and Parasuraman, 2010), among many other characteristics. Automation may also handle a large variety and complexity of tasks, in many different environments, some of which may be interpreted as more or less appropriate for automation (Hertz and Wiese, 2019) and thereby influence trust.

Regardless of these variations in design, TiA only becomes relevant when the human is uncertain that their teammate will perform competently and reliably and when risks are tied to that performance. Without either, trust is replaced with certainty and control (Das and Teng, 2004). Prior research has introduced uncertainty and vulnerability in a variety of ways with a variety of tasks, such as pasteurization controllers with performance bonuses at stake (Lee and Moray, 1994), health decision aids with patient health at risk (Pak et al., 2012), or convoy route planning aids with the risk of a simulated attack (Lyons and Stokes, 2012). In these scenarios, the operator must determine whether the automation is worthy of their trust, given that failure and harm are possible whether they rely on the automation or rely on themselves. Given the uncertainty and vulnerability involved in trusting an automated teammate, it is crucial for the operator’s trust to be properly calibrated, neither trusting the automation too much nor too little for the situation and its ability (Lee and See, 2004). Mis-calibrated trust between humans and automation may lead to misuse or disuse of automation, each with their own set of consequences to safety and efficiency (Parasuraman and Riley, 1997).

Capturing the current level of trust is not a simple task: Trust is commonly conceptualized as a latent variable that cannot be directly observed but rather must be inferred. Trust is dependent on the interplay between analytic, analogical, and affective processes, especially emotional responses to violations or confirmations of expectations (Lee and See, 2004). These affective responses mean that trust is not solely a cognitive process, but also an emotion which varies over time (Fine and Holyfield, 1996). Trust can vary due to a variety of factors, including factors of the automated partner, the environment in which the task is occurring, and characteristics of the human interaction partner (Schaefer et al., 2016). Furthermore, the nature of trust itself changes during an interaction, shifting rapidly from trust due to the disposition of the human interaction partner to trust due to interaction with the automation (Merritt and Ilgen, 2008; Hoff and Bashir, 2015).

In consequence, TiA can be difficult to accurately measure. Likert-type scales employ an ordinal indicator of the participant’s trust in the automation, but these scales abridge trust into a simple value that may not properly capture the contextual nature of trust, its fluidity over time, or external biases. If we envision trust as a process, as proposed by Mayer et al. (1995), then the dynamic nature of trust is even less well-suited for capture by infrequent Likert scales. Some authors suggest that trust may be context- and task-specific (Lewicki et al., 1998) and that trust and distrust can exist simultaneously (Lewicki et al., 2006). These arguments further complicate the waters of self-report trust measurement. Despite these difficulties, it is crucial for designers and researchers to be able to measure TiA. Accurate measurement can give context to user behavior and help direct the design of automation, yet researchers currently lack a reference work/educational tool that empowers them to pick trust measures for their research and understand precisely what is being captured by each measure. The resulting review consists of:

1. A constructive inventory of prior TiA research’s measurement of trust;
2. A synthesis of existing trust measurement methods that examines which component of trust from Mayer et al.’s (1995) trust model is being measured by each method; and
3. A discussion and critique of TiA measurement and potential solutions to identified deficits.

To grant greater context to these topics, we provide an overview of Mayer et al. (1995) process-oriented model of trust and the approach that was taken to survey existing methods of trust.

### Models of TiA

#### Background

The empirical measurement of human trust is relatively new, even within behavioral psychology. One of the first such measurements captured perceptions of the ethos (including trustworthiness as a character trait) in humans delivering a speech (McCroskey, 1966). Scales of interpersonal trust were developed and began to be widely used in the 1970s and 1980s (Rotter, 1971; Rempel et al., 1985). The concepts of trust in computers and automation promptly manifested when those concepts became widespread. While mechanical automation has been around for centuries (i.e., temperature regulators invented by Cornelis Drebbel in the 1620s and electromechanical analog fire control computers in the second World War), the rise of personal computers in the 1980s began to expose exponentially more people to increasingly capable and complex automation. This automation was more flexible than the highly specialized automation of earlier eras, and – as a consequence of greater uncertainty and vulnerability associated with the use of these devices – required greater trust from its users. TiA is relatively similar to trust in other humans, in that both represent a situation-specific attitude that is relevant when something is exchanged in a cooperative relationship characterized by uncertainty (Hoff and Bashir, 2015). While the differences may seem subtle, they are crucial and manifold, affecting the inception of trust through to its evolution and recovery (Madhavan and Wiegmann, 2007; de Visser et al., 2016). Therefore, there is a strong need for empirical trust research specific to automation, which began in earnest with seminal works by Muir (1983, 1987) and Lee and Moray (1991). As the quantity of research on this topic increased, so did the capability to generate mature and informed models and conceptualizations of TiA.

Conceptualizations of trust have become increasingly unified in recent decades, but there is no single universally accepted definition or model of TiA, perhaps due to the contextual nature of trust. Lack of a unitary model means that the concept of “trust” being measured may vary based on the intent and conceptualization of the individual researcher – assuming said researcher has couched their measurement within a model at all. This is understandable, given that most models of trust do not sufficiently specify how trust measurement is related to the overall model or its components. The disconnect of measurement and models prevents systematic scientific inquiry of the trust topic. For example, the highly influential and useful Lee and See (2004) and Hoff and Bashir (2015) models do not relate their conceptual models explicitly to different types of trust measurement. Meta-analyses for human-automation trust (Schaefer et al., 2016) and human-robot trust (Hancock et al., 2011) do relate to measurement as a consequence of requiring trust measures in the studies that inform their models. Yet even in those analyses, it is not always apparent which measures were used, because the results of multiple studies are presented in the aggregate. As a result, trust theories cannot be sufficiently tested, because it is unclear which measures are appropriate to test the specific hypotheses derived from these theories. Similarly, a lack of clearly defined measures as they connect to trust theory has also forced scientists to create their own *ad hoc* measures that capture trust as a monolith, rather than a targeted aspect of trust theory. As a result, it is often unclear how a given study’s results contribute and fit into the larger story about TiA. This may duplicate efforts, wasting the time and resources of scientists interested in exploring trust because there is no coherent narrative and no clear “state of the art” for TiA research. Exceptions do exist to this critique: Some recent measures such as the TOAST (Wojton et al., 2020) and multi-dimensional measure of trust (MDMT) (Malle and Ullman 2021) explicitly and intentionally relate to existing models. These measures are some of the few exceptions that prove the general rule.

To frame our present discussion of trust measures and to directly address the above critiques, we are focusing on Mayer et al.’s process-oriented model of organizational trust (1995). While Mayer’s model was not originally intended for automation, it is a commonly used model of trust (Rousseau et al., 1998) and has therefore been adopted to TiA as well (see Lee and See, 2004, for a discussion of said adaption). Discussing trust measurement within the context of a model that is often referenced in automation literature enables our analysis to delineate exactly which component of trust is being captured by each measure and to grant a consistent and familiar meaning to each measure. No single trust model cleanly envelopes all of the available trust measures, but Mayer’s model is popular and comprehensive, and the process-oriented nature of the model enables us to directly relate trust measures to different aspects of the trust process. This effort will assist practitioners in understanding what is being captured by each trust measure, what factors may influence the outcomes of each measure, and explain variations within these outcomes. Agnostic of models, trust is a complex combination of constructs, and many measurement methods capture a facet of trust rather than the entire concept – even if the measure’s creators did not originally draw that distinction. Thus, any discussion of trust measures must be intelligibly couched within the context of these models.

#### Trust Model

**Figure 1:**
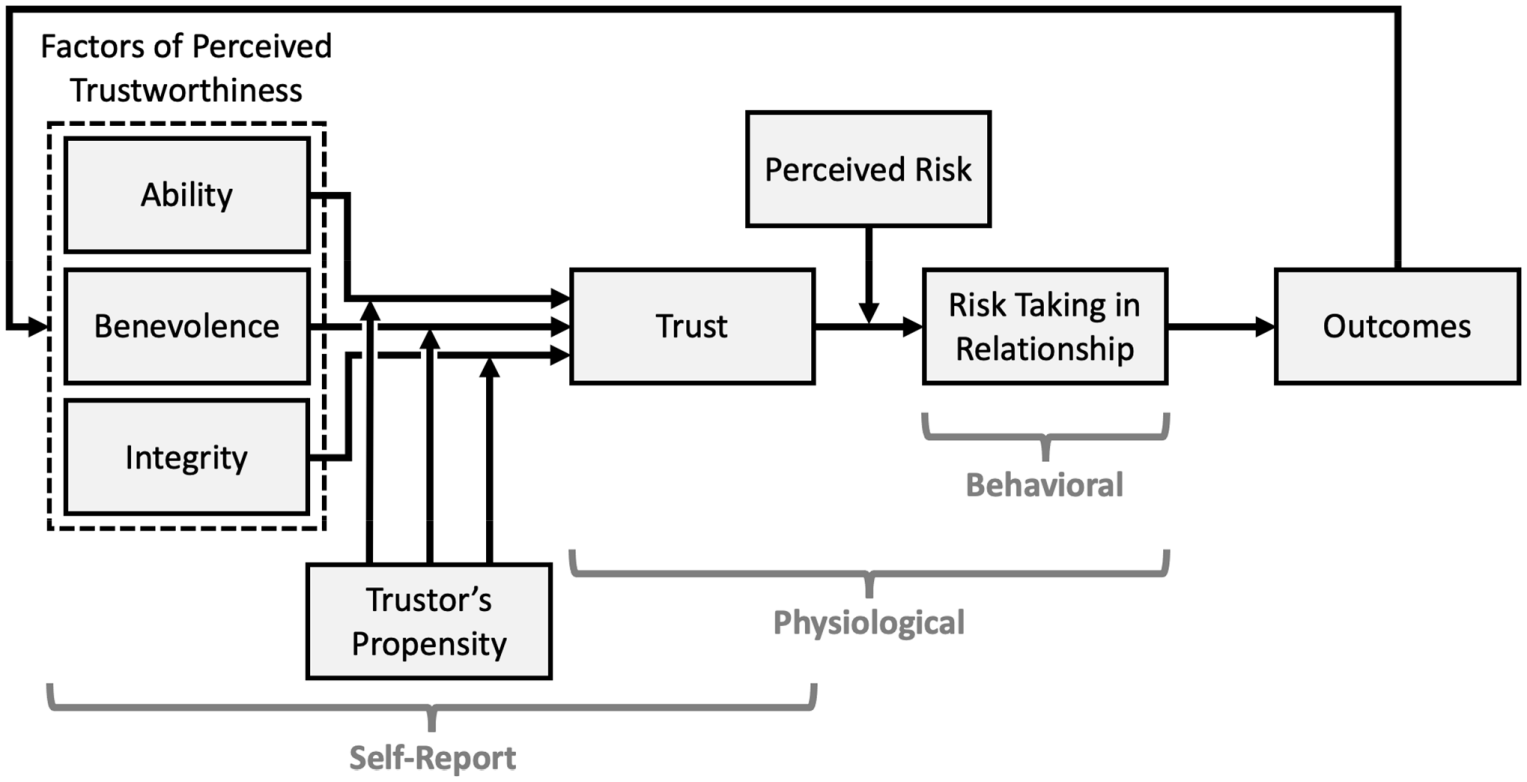
An annotated reproduction of Mayer’s organization model of trust (Mayer et al., 1995) noting which measurement methods (labeled brackets) are typically used to capture different constructs (rectangles) of the model.

Mayer et al. (1995) created a process-oriented model of trust that has been widely adopted by the TiA community despite its original intent as a model of organizational trust (Mayer et al., 1995). In part, this adoption may be due to the clear differentiation between six primary components of trust. While the model at large is ostensibly a trust model, only one of the six components is trust itself – the others are antecedents, context, and products of trust. These components are not only crucial for understanding trust as an attitude and behavior, but also establish the uncertainty and vulnerability cited in Lee and See’s (2004) trust definition. Furthermore, while trust measures often do not self-identify as capturing a specific component of any given model, we have found that many trust measures fit cleanly within one or more of the components defined by Mayer. To persuasively communicate this argument, we will describe each component within this model and relate how they could theoretically be captured by different measurement methods. Figure 1 broadly displays which categories of trust measures capture which trust model components, while Table 1 provides a granular breakdown of how each component may be measured.

**Table 1:** Comparing trust models to process and measures.

Trust type	Trust process step	Measure category	Experiment step
Factors of perceived trustworthiness	The perception of the system’s trustworthiness-related characteristics	Self-report	Before/during interaction
Trustor’s Propensity	Effects of the individuals’ traits (culture, genetics, and age)	Self-Report	Before Interaction
Trust	The trust stance or attitude that exists during interactions, continually influenced and updated by feedback	Self-Report; Physiological	During/After Interaction
Perceived Risk	Effects of the individual’s understanding of the situation, including risk	N/A	Pre-Interaction, in environmental situation
Risk-Taking in Relationship	The trust behavior that is expressed during interactions, continually influenced and updated by feedback	Behavior	During/After Interaction
Outcomes	The combination of system accuracy and user trust, that provides feedback and influences future trust attitudes	N/A	During/After Interaction

##### Trustworthiness

The first component of Mayer’s model is the three Factors of Perceived Trustworthiness: ability, benevolence, and integrity. Ability is skills and competencies; integrity is the degree to which the trustee adheres to a set of principles that are acceptable to the trustor; and benevolence is the extent to which the trustee’s motivations are aligned with the trustors. Notably, this first component is the one that translates least cleanly between automation and the original organizational focus of this model. Both automation and humans possess ability, though automation suffers from some assumptions in regard to its latent ability (Automation Bias, Parasuraman and Riley, 1997; and the Perfect Automation Schema, Dzindolet et al., 2002). The same is not equally true for integrity and benevolence, and prior research suggests that some agents may not be perceived as possessing these qualities. See Malle and Ullman (2021) for a discussion of moral trust in agents.

Lee and See (2004) have provided an automation-friendly translation of these three factors: performance (how well the automation is performing), process (in what manner and with which algorithms is it accomplishing its objective), and purpose (why the automation was built originally). Regardless of the iteration used, this component of the trust model captures perceptions of the system’s trustworthiness-related characteristics prior to, or during, interaction. The primary method of capturing these perceptions is self-report – asking participants to answer survey questions concerning their assessment of the agent’s ability, integrity, and benevolence. While some surveys explicitly capture these three factors (Mayer and Davis, 1999), most capture a set of dimensions which obliquely include these factors in addition to other trustworthiness queries: For instance, the Checklist for Trust (Jian et al., 2000) has questions that closely map to ability, integrity, and benevolence, in addition to others which address trustworthiness in a more oblique manner. Other surveys focus on trustworthiness as a singular construct without component factors (Lee and Moray, 1992, 1994). Notably, the predictive effect that ability, benevolence, and integrity perceptions have on trust changes over time with accumulated experience (Serva et al., 2005), making repeated measurement of these component factors crucial.

##### Trustor’s Propensity

Participants’ perceptions of ability, integrity, and benevolence (and thus trustworthiness at large) are influenced by their innate propensity to trust – static traits caused by culture, experiences, and personality types that moderate how perceptions of trustworthiness translate to trust attitude. Propensity to trust is stable over time and impacts whether a trustor will trust their partner before they have shared any experience: Trustors enter the relationship with a pre-established conception of the trustee’s trustworthiness. This mental schema is most often captured by self-report measures. Notably, the effect of trustors’ propensity has a waning effect as the trustor accumulates experience with the trustee, which may be captured by measuring trust before and after the trustor has established a history of interaction (Merritt and Ilgen, 2008).

##### Trust

Only one of the six primary constructs of Mayer’s trust model is trust itself. Within this model, trust is a cognitive stance or attitude that exists during interactions: the theoretical willingness to engage in trusting actions. As such, while it may be inferred from the trustworthiness perceptions that precede it or from the behavioral expressions of trust that proceed from it, trust itself is a distinct construct. Consequently, measurement of trust attitudes is often conflated with measuring these accessory constructs. Appropriate methodology can attempt to capture a cleaner sample of trust attitudes *via* self-report or physiological methods, but both have distinct weaknesses. Asking trustors to self-report their own level of trust is extremely common within TiA research (Hancock et al., 2011), yet establishing construct validity is difficult for such latent variables. Trust measurement *via* physiological methods may avoid self-report’s issues with introspective (in-)ability by directly capturing trustors’ internal calculus. Unconscious mental calculations of conditional and unconditional trust (Krueger et al., 2007), and moral character (Delgado et al., 2005), and reputation (King-Casas et al., 2005) are reflected in neural activation, yet the promise of neurological measurement has not been substantially leveraged to capture TiA at this time.

##### Perceived Risk

As previously established by Lee and See’s definition of trust (2004), vulnerability is a crucial component of trust; trust in a situation that has no potential for negative consequences is effectively meaningless. The potential for said consequences is risk, which moderates the relationship between a theoretical willingness to trust versus trusting behaviors (Mayer et al., 1995). Accordingly, individuals are less likely to engage in trusting behaviors in situations where they perceive high risk (Ezer et al., 2008; Lyons et al., 2011; Satterfield et al., 2017). Given that risk is a situational moderator that is unaffected by perceptions of trustworthiness and unchanged by feedback, direct measurement of this construct is out of scope of the current review.

##### Risk-Taking in Relationship

Trustors balance the strength of their trust attitude with the situational risk they perceive. If trust outweighs risk, then the aforementioned theoretical willingness to trust will lead to a behavioral expression of trust: increased risk-taking in the relationship (Mayer et al., 1995; Colquitt et al., 2007). The trustor will behaviorally rely on the trustee in a manner that makes themselves vulnerable in hopes of supporting and meeting their goals. Risk-taking does not require trust to occur; individuals may take risks solely based on a probability-based gamble, but more risks will be taken with the trustee after trust is established. As risk-taking is a behavior – typically taking the form of reliance or compliance with the trustee – it may be captured and used to estimate trust states.

##### Outcomes

The trustor’s risk-taking interacts with the trustee’s reliability to produce an outcome. The value of that outcome – whether quantitative or qualitative, positive or negative – is assessed by whether it helped or hindered the trustor’s goals. This assessment is the primary feedback that influences future trust attitudes, informing evaluations of ability, benevolence, and integrity. These updated trustworthiness perceptions power the next cycle of trust and feedback. Thus, trust gradually develops from a mental calculus based on face values to trust based on experience and feedback (Lewicki et al., 2006). While outcomes are a partial product of the trustor’s risk-taking, they are generally viewed as independent from trust itself and are not utilized as a trust proxy.

#### Relating Trust Measures to the Model

Mayer’s process-oriented model provides a clearly defined context for discussing trust measurement, as it delineates trust from its antecedents, context, and products. Such borders allow us to declare that a given measure is capturing a specific dimension or facet rather than a monolithic “trust.” Such an effort would help to better understand what is being measured, ensure the validity of these measures, and help explain discrepancies or variations within and across trust measurement. Toward this end, our prose breakdown of Mayer’s model defines each construct, Table 1 decomposes the trust process, and Figure 1 visually presents the model. The constructs discussed here will be used to contextualize the trust measures presented in this review, contrast their products, and propose methodological improvements.

## Methodology

### Approach

This work follows the integrative narrative review methodological approach defined by Pautasso (2013) and outlined by Torraco (2005), wherein the trust measurement methods in question are inventoried and their value qualitatively – rather than quantitatively – compared. Each measure is mapped to Mayer’s integrative model of organizational trust (Mayer et al., 1995). A direct quantitative comparison of different methods of measuring TiA is impractical due to the varying purposes, timing, and components of trust being measured. Therefore, while this review will not dictate which measure is best, our inventory will explain how each trust measure is applied, as well as which constructs of trust are captured by each measure.

### Keywords

The authors iteratively developed a set of keywords to capture the maximum quantity of relevant literature. The core concept keywords were “Trust AND Automation OR Autonomous,” which represented the concepts of interest. Keywords surrounding robotics were excluded to constrain the scope of this work and limit irrelevant works. This constraint is acceptable given that (1) automation may be embodied in a robot, but robotics is not definitionally automated; and (2) works concerning automated robots were included *via* our supplementary reference search and subject matter experts (SME) solicitation as explained below. Specific search keywords were added to these core concepts. These specific keywords were based on exemplar TiA papers, in addition to any keywords that were subjectively determined to be relevant.

Searches strings were initially iterated within the PsychINFO and ACM databases, with subjective assessment confirming that the search results included relevant works and that the original exemplar papers were included in the search results. Collection criteria were limited to journal articles, conference publications, and early access works published on or after 1970, with a publication cutoff of June 2020. Only the Booleans “AND” and “OR” were used. The resulting 37 search strings each included “Trust AND Automation OR Autonomous” and a specific keyword added with an “AND” Boolean. The keywords used are listed in Table 2, and the databases utilized are as follows: PsychINFO, ACM, and IEEE. These databases were selected for their range of peer-reviewed works and the stable, replicable nature of searches performed there. Searches were performed using the broadest possible advanced settings in each database.

**Table 2:** Keywords used.

Core concept	Keywords, used following “AND”
Trust	Subjective, Confidence, History, Disposition, Forgiving, Avoidance, Revenge, Reciprocity, Reliance, Compliance, Complacency, “Take-over,” “Take over,” Allocation, Allocate, Verification, Verify, Dependence, “Eye-Tracking,” “Eye Tracking,” Neural, Brain, Cognitive, Neuroimaging, Neuroeconomics, Neuroergonomics, Neuroscience, MRI, fMRI, ERN, ERP, Pe, tCDS, Oxytocin, Cortisol, Hormones, Peptide, Neuropeptide
AND Automation OR Autonomous	

### Selection Criteria

The searches were performed in June 2019 – with follow-up searches in June 2020 to capture 2019–2020 publications – and produced 41,923 results: 16,569 from PsycINFO, 6,458 from ACM, and 18,896 from IEEE. Due to the intentionally broad nature of the initial keywords and searches, the majority of the resulting works were irrelevant and were iteratively excluded using the selection criteria below. To limit subjectivity, works that could not be concretely excluded were jointly assessed by authors SCK, EJdV, and THS to determine whether they met the inclusion criteria. The criteria are as follows:

- The work was empirical;
- The work was available in English;
- The work concerned the interaction of humans and automation or autonomous systems;
- The work intended to measure the human’s TiA or autonomous systems;
- The work reported significance testing (frequentist or subjectivist) for the trust measure.

To maximize the completeness of our works, we also performed a secondary set of inclusions. The reference lists of three exemplar works (Hoff and Bashir, 2015; Schaefer et al., 2016; de Visser et al., 2020) were reviewed to find papers that were not included in the initial database searches. This process uncovered 27 additional papers which adhered to inclusion criteria. TiA SMEs was solicited *via* e-mail for any works that they determined were relevant. Four SMEs responded, providing more than 300 relevant works, many of which were redundant with the existing list of publications. Notably, several of the exemplar work and SME-recommended trust measures were developed for use with robots, rather than non-embodied automation. Due to these measures’ flexibility and applicability to automation, we included these works under the condition that they had been at least once applied to measure trust in non-embodied automation as well.

### Exclusions

This review is potentially limited by several factors related to which sources were sampled. The scope of the sample was restricted to works concerning automation or autonomous systems and did not include “machine” or “robot” as keywords as those keywords exponentially increased the count and irrelevance of results: Machines and robots may be automated or autonomous but are not required to possess that characteristic. Regardless, some trust in robot surveys were included in our review for reasons previously referenced. Our search terms were also based on search terms within known publications. The searches themselves are subjected to the limitations of the databases used, which may not include all preferred publications. The reference list review and SME solicitation are intended to resolve such gaps. In consequence, we are confident that the current review is proportionately representative of how TiA is typically measured.

## Measures of TiA

Thirty measures of trust, applied to TiA, were uncovered in this review. These measures spanned self-report, behavioral, and physiological measures of trust. Each measure has been applied to TiA, though not all were originally intended for this narrow trust context – some measures were originally developed to capture interpersonal trust or trust in robots or autonomous systems. These measurement methods capture several different constructs of trust as defined by Mayer and delineated in Figure 1 and Table 1, respectively. The adjudication for mapping each measure onto a relevant construct from Mayer’s trust model (1995) was performed by the authors of this review, except when the creators of each measure explicitly identified the construct the measure intends to capture. Those few cases are discussed in the description of each measure. The measures themselves are described below and listed in Tables 3–5.

**Table 3:** Self-report measures of trust.

*Measure*	Originating Source	Relevant construct from Mayer et al. (1995)
*Checklist for Trust*	Jian et al. (2000)	Factors of Perceived Trustworthiness; Trust
*Complacency Potential Rating Scale*	Singh et al. (1993)	Trustor’s Propensity
*Custom Scales*	None	Trust
*Dynamic Reporting of Trust*	Desai (2012)	Trust
*Human-Computer Trust (HCT) Questionnaire*	Madsen and Gregor (2000)	Factors of Perceived Trustworthiness; Trust
*Individual Differences in Trust in Automation*	Varied	Trustors’ Propensity
*Integrated Model of Trust*	Muir and Moray (1996)	Trust
*Measures of Trust & Trustworthiness*	Mayer and Davis (1999)	Factors of Perceived Trustworthiness; Trust; (Propensity & Outcomes not frequently used)
*Multi-Dimensional Measure of Trust (MDMT)*	Malle and Ullman (2021)	Perceived Trustworthiness; Trust;
*Self-Reports of Automation Qualities (Reliability, Accuracy)*	None	Factors of Perceived Trustworthiness
*Trust & Self-Confidence Scale*	Lee and Moray (1994)	Trust
*Trust in Automated Systems Test (TOAST)*,	Wojton et al. (2020)	Factors of Perceived Trustworthiness
*Trust in Automation Scale*	Körber et al. (2015)	Factors of Perceived Trustworthiness; Trust
*Trust Perception Scale for Human-Robot Interaction (HRI)*	Schaefer (2016)	Factors of Perceived Trustworthiness; Trust
*Trust Scale, Lee & Moray*	Lee and Moray (1992)	Trust
*Trust Scale, Merritt*	Merritt (2011)	Factors of Perceived Trustworthiness; Trust

**Table 4:** Behavioral measures of trust.

*Measure*	Representative source	Relevant construct from Mayer et al. (1995)
*Combined Team Performance*	de Visser et al. (2016)	Outcomes
*Compliance/Agreement Rate*	Chancey et al. (2017)	Risk-Taking in Relationship
*Decision Time*	Yuksel et al. (2017)	Risk-Taking in Relationship
*Delegation*	Xie et al. (2019)	Risk-Taking in Relationship
*Economic Trust Game: Stakes Invested*	Berg et al. (1995)	Risk-Taking in Relationship
*Intervention*	Itoh et al. (1999)	Risk-Taking in Relationship
*Reliance*	Rice (2009)	Risk-Taking in Relationship
*Response Time*	Körber et al. (2018)	
*Verification*	Ho et al. (2005)	Risk-Taking in Relationship

**Table 5:** Physiological measures of trust.

*Measure*	Representative source	Relevant construct from Mayer et al. (1995)
*Electrodermal Activity*	Akash et al. (2018)	Trust; Perceived Risk
*Eye Gaze Tracking*	Hergeth et al. (2016)	Trust
*Heart Rate Change & Variability*	Waytz et al. (2014)	Trust; Perceived Risk
*Neural Measures*	de Visser et al. (2018)	Factors of Perceived Trustworthiness; Trust

### Self-Report Measures

The self-report measures are defined as measures in which respondents report on their own behaviors, beliefs, attitudes, or intentions by receiving a question or prompt and selecting a response. Surveys and questionnaires are the typical method of self-report. Many such measures utilize Likert or sliding scales, which require respondents to report these qualities along an ordinal or interval scale. Sixteen different types of self-report methods for capturing TiA are identified below in Table 3 and are defined in more detail below in alphabetical order. The origin of each measure is reported in the table.

#### Checklist for Trust Between People and Automation

The most frequently used self-report measure of TiA measurement with a cited source is inarguably Jian et al. Checklist for Trust between People and Automation (2000) (Jian et al., 2000). This scale is distinct in that it measures trust and distrust as polar opposites along a single dimension rather than simple unidimensional trust. Therefore, the output may be a single all-encompassing trust value or separate values for the trust and distrust dimensions. This 12-item set of Likert scales includes a variety of items sampling distrust, such as perception of the automation’s deceptive nature or the likelihood of harmful outcomes if it is used. These items must be reverse-coded if used to create a singular trust score. The trust items on the scale include assessments of reliability, integrity, and overall trust. Several of these items are conceptually similar to the Factors of Perceived Trustworthiness defined by Mayer et al. (1995). Due to the length of the survey, this survey is typically deployed only after each experimental block or at the end of the task.

#### Complacency Potential Rating Scale

The seminal complacency scale developed by Singh et al. (1993) captures complacency toward automation, characterized by whether an operator is likely to ignore automation based on the belief that the system is, and will remain, in a satisfactory state (Singh et al., 1993). The 20-item survey captures attitudes toward automation in general and three items capturing trust. Similar to dispositional trust surveys, this measure captures trust prior to interaction and therefore excludes the influence of trust learned or calibrated through interaction with the automation. Similarly, this scale may be administered prior to almost any type of task.

#### Custom Scales

The most frequently used method of TiA measurement – cited or otherwise – is custom self-report scales. These scales are distinct in that they either explicitly identify themselves as a custom creation or simply did not cite a source. In some instances, these scales cite inspirational sources, but in those instances, the end product scale is modified beyond the original sources to a degree that it can no longer be attributed to any origin scale.

Custom measures ranged widely in intent, labeling, number of measures, and placement within the experimental task. As such, they are not identified as collectively representing a component or components within the Mayer et al. (1995) trust model. However, the vast majority of these custom creations are Likert or sliding scales, often with only a single item. This single item was typically a theme on “To what degree do you trust [this autonomous/automated system]?.” Despite the lack of citation, this item is functionally similar to the trust item used in Lee & Moray’s trust and self-confidence measure (1994): “How much did you trust the automatic controller of the steam pump?.” The overlap is unlikely to be intentional in most instances. Due to the typically parsimonious nature of these custom scales, they were easily integrated into many different types of tasks and often repeated throughout the task.

#### Dynamic Reporting of Trust

Dynamic reporting of trust fills a particular niche in the self-report measures, enabling participants to respond at extremely frequent intervals with minimal interruption to the ongoing task (Desai, 2012). Responding at a particular cue or at will, participants indicate whether their trust has increased, remained the same, or decreased compared to their prior response (Desai, 2012; Desai et al., 2013). Therefore, trust is measured relative to the prior report rather than relative to a static scale, which mandates repeated sampling to make the output truly meaningful. Some of these measures were deployed in a fashion that enabled participants to freely respond at the moment that they were aware of their own trust changing. While this minimalistic sampling method could be applied to most types of experimental tasks, the mandate to sample frequently means that this measure should only be deployed when near-real-time trust is required and can be analyzed to preserve its temporal specificity.

#### Human-Computer Trust Questionnaire

The human-computer trust (HCT) questionnaire developed by Madsen and Gregor (2000) focuses on capturing trust in computer systems, specifically artificially intelligent decision aids (Madsen and Gregor, 2000). Five facets of trust (perceived understandability, perceived technical competence, perceived reliability, personal attachment, and faith) are captured using five items each, for a total of 25 survey items. While the score for each facet may be used to analyze trust in detail, an average score across all 25 items is often used to represent overall trust.

#### Individual Differences in TiA

Perception of a teammate’s trustworthiness is influenced in Mayer’s model by the operators’ innate propensity to trust. This is distinguished from all other factors within Mayer’s model, in that it is solely the product of the operator’s disposition, not their history of interaction with the teammate. There is a wide range of disposition components that may be captured using self-report TiA measures, including the following: operators’ tendency to be complacent toward automation (Automation-Induced Complacency Potential Rating Scale, Singh et al., 1993); operators’ high expectations for the automation (Perfect Automation Schema, Merritt et al., 2015); and operators’ tendency to trust automation (Merritt and Ilgen, 2008). There is also substantial evidence that personality influences TiA (Szalma and Taylor, 2011). All of the cited surveys have been frequently used to contextualize TiA behaviors. As they are administered prior to interaction, these measures may be applied to almost any type of task without disrupting the experiment. One of the most common surveys is Merritt and Ilgen’s six-item capture of dispositional trust (2008), which is designed to be administered before and after the experiment. In doing so, it captures both trustors’ propensity and trust, enabling comparison of trust due to individual differences and trust influenced by a history of interaction.

#### Integrated Model of Trust

Muir and Moray’s prior trust model (1994) set the stage for a simple two-item measure of trust proposed in 1996 (Muir and Moray, 1996). This survey had a single item capturing trust on a 100-point scale and an item that captured the participant’s confidence in their own rating of trust. While the measure was intended for use with automated factory plant machinery, it was also used to capture trust in other types of non-automated systems. Potentially due to the fact that the confidence item simply qualifies the trust scale rather than providing any unique measure of the automation, many researchers who chose to implement this survey chose to omit that item in favor of the trust item alone.

#### Measures of Trust and Trustworthiness

Mayer’s trust model (1995) decomposed trust into several distinct constructs, which were later captured by Mayer and Davis’ (1999) survey (Mayer and Davis’ 1999). This survey separately captured ability, benevolence, integrity, propensity, trust, accuracy, and outcome instrumentality, enabling a direct comparison to the original trust model. Notably, only the measures of trust and trustworthiness have been widely used within TiA, often modified to fit the automation context (e.g., the use of Mayer’s trust-specific measures in Lyons and Guznov, 2019).

#### Multi-Dimensional Measure of Trust

The MDMT is a multi-dimensional measure of trust, designed to capture trust in robots, rather than automation (Malle and Ullman, 2021). However, the authors explicitly identify the performance dimension of trust as being central to human-automation trust, while the moral aspect is more aligned with robots. This measure is particularly apt as it recognizes that not all dimensions will be applicable to all agents nor all interactions – the MDMT enables participants to decline to answer any of the 16 questions if they are not applicable to the agent. This flexibility makes this survey more aptly convertible to human-automation trust.

#### Self-Reports of Automation Qualities (Reliability, Accuracy)

Self-reports of the participants’ perception of the automations’ competence have been used to infer trust. While these methods have no specific cited source nor identical wording, these self-reports (e.g., in Wiegmann et al., 2001; Yuksel et al., 2017) capture a family of concepts related to automation competence. These typically single-item surveys asked participants to evaluate the automation’s reliability or accuracy and used those subjective results in turn to infer the psychological construct of automation trust. While we caution against solely using these competence-based survey items to capture trust, these queries are similar to the ability construct identified within Mayer’s Factors of Perceived Trustworthiness (1995) or the predictability and dependability items used within Lee and Moray’s, 1992 trust scale.

#### Trust and Self-Confidence Scale

A set of paired questions regarding trust in the partner performing the task and self-confidence in performing the task provides greater context for trust and reliance/compliance decisions in Lee and Moray’s (1994) measure (Lee and Moray’s (1994). Taken together, these metrics proport to predict allocation strategy with an automated teammate: If trust exceeds self-confidence, the operator will rely on the system. This context is crucial, given that reliance does not always change in response to changes in trust (Lee and Moray, 1992). This lack of change may be due to differences in self-confidence.

#### Trust in Automated Systems Test

The Trust in Automated Systems Test (TOAST) scale developed by Wojton et al. (2020) captures two dimensions of trust: understanding the system and system performance (Wojton et al., 2020). The scale was tested for model fit and validated against existing measures of trust. The resulting nine survey items represent these two proximate causes of trust, conceptually similar to the Factors of Perceived Trustworthiness (Mayer et al., 1995).

#### TiA Scale

The TiA scale developed by Körber et al. (2015) captures five subscales (Reliability/Competence, Familiarity, Trust, Understanding, and Intention of Developers) containing between two and four items, for a total of 19 items Körber et al. (2015). Each facet may be analyzed independently, and prior studies have focused on the reliability and trust facets. A calculated score of all 19 items may also be used to represent overall trust. The original scale is in German, yet has been translated for English language applications by the original authors (see supplemental materials in Körber, 2018, or access directly at https://github.com/moritzkoerber/TiA_Trust_in_Automation_Questionnaire).

#### Trust Perception Scale for Human-Robot Interactions

The trust scale developed by Schaefer (2016) is notable for several reasons (Schaefer, 2016). As implied by the title, this scale is developed for interaction with robots, rather than generic machines or automation. While the scale has been used with automation, some of the items may be more suited to embodied robots than software alone. Schaefer provides a 14-item sub-scale within the larger scale that happens to be more appropriate for automation.

Both scales bypass the traditional method of asking the participant to self-report their own attitudes and instead capture trust by asking participants to estimate percentages of the time that a given robot will meet specific criteria or possess certain attitudes of its own. Finally, this scale is relatively unique in that it is intentionally designed to capture trust before and after interactions, capturing both initial dispositional trust that is the focus of Merritt’s surveys (e.g., 2008) and trust that is learned *via* interaction with the system (see: Hoff and Bashir, 2015 for a discussion of learned trust). While most self-report scales can capture those facets of trust, few are designed with the explicit intent of pre-post interaction trust capture. Due to the substantial length of this survey, the full 40 items are ideally administered pre-post interaction and not during the experiment. The same limitation applies, to a lesser degree, to the 14-item scale.

#### Trust Scale

Lee and Morays trust scale was based on prior work by Muir (1989) and captures predictability and dependability of the automation system as well as faith and trust in the system, with one item per dimension (Lee and Moray, 1992). These four items in combination represent the larger construct of trust, and indeed, the individual items for predictability and dependability mirrored the results for the independent trust item. Perhaps for this reason, many researchers who deployed this trust scale used only the single trust-specific item.

#### Trust Scale

Merritt’s trust scale (2011) was a custom creation that captures both the general construct of trust, but also the trustworthiness components of ability and benevolence per Mayer’s model (1995) (Merritt, 2011). These six Likert-scale items are readily adapted for measuring TiA and have been used or adapted periodically in other studies.

### Self-Report Discussion

Self-report is a traditional and well-established method of capturing participants’ attitudes and beliefs in both interpersonal relationships and in interaction with machines. These measures seek to capture the participant’s perception of the automation’s ability, integrity, and benevolence (Mayer’s Factors of Trustworthiness, 1995), as well as their perception of their own Propensity to Trust and their internal Trust attitude. The latter is, of course, a latent variable that is difficult to directly measure or for a participant to precisely estimate. These three constructs are captured *via* surveys, some of which are precise in targeting and measuring a given construct, whether by intentional or by coincidental mapping to trust models. However, many trust surveys indirectly capture a single construct or a blend of multiple constructs and identify the result as being the monolithic construct of trust. Despite these potential issues, there are a large quantity of trust surveys of varying qualities. Their pros and cons are discussed here, as well as a larger critique of various approaches used by these surveys. A discussion of popular measures and their quality is also provided.

#### Critiques, Considerations, and Recommendations for the Use of Self-Report Measures

The trust self-report measures available to researchers range from the extremely brief (e.g., Desai’s (2012) stepped measure or Lee and Moray’s (1994) two-item trust and self-confidence survey) to very thorough (Madsen and Gregor’s, 2000 25-item HAT survey). Self-report measures are extremely easy to integrate into existing tasks, often with mild intrusiveness and little alteration of the existing task. Moreover, self-report measures of trust, whether unidimensional or multi-dimensional, tend to have better face validity as the measure has been intentionally developed to capture trust as a construct. Due to these reasons, they can be applied to nearly any experimental task in a variety of contexts.

While self-report is the most often used measure of trust, it is not without its limitations. First, administration of the scales requires interruption of the task, which can alter the nature of the task and lead to performance decrement (Moroney et al., 1995). Furthermore, as self-report generally requires pausing the task, the surveys are often infrequently administered and therefore may not capture the full evolution of trust. The self-report scale may alternatively be administered after the task has been completed, but it is then subject to memory failures and operator bias.

Second, self-report results do not consistently and perfectly align with actual trust behavior. These measures are correlated and sensitive to changes in automation reliability but are not well calibrated with each other (Wiegmann et al., 2001). A portion of this discrepancy may be due to self-report and behavioral measures capturing different components of trust, yet both measure types are likely subject to biases and do not perfectly capture their intended construct.

Third, recent analysis has uncovered some weaknesses in popular self-report measures of trust. In particular, the Checklist for Trust (Jian et al., 2000) has been found to have less sensitivity than other measures (Schaefer, 2016) as well as a positivity bias (Gutzwiller et al., 2019). Further questions have been raised about whether the underlying trust-to-distrust dimension underpinning this scale is accurate or whether trust and distrust are orthogonal. Prior work has explored trust and distrust as a two-dimensional construct versus the default unidimensional concept (Lewicki et al., 2006), while some research has found that trust and distrust load onto different factors (e.g., Chien et al., 2014). Some authors have suggested that distrust may be independent and more sensitive than trust-based scales (Tenhundfeld et al., 2019). Jian’s checklist has drawn particular focus and critique due to its overwhelming popularity within TiA (Gutzwiller et al., 2019; Kohn et al., 2020), but these critiques are not unique to this scale. Researchers should be cautious when deploying self-report measures and ensure that the scales they are using have been previously validated and are appropriate for the given task and analysis goals. Despite these limitations, the ease, accessibility, and relative accuracy of self-report measures have fueled their ubiquity. For many researchers and tasks, self-report may be the best option, yet there is substantial room for improving the measures and their application.

In reviewing the self-report measurements in use, we found that custom measures were extremely common – more so than any other form of trust measurement, self-report, or otherwise. As insinuated in the introduction, the prevalence of custom measures may be an artifact of researchers not knowing what methods are available to them. However, some of these custom measures were likely created because the most readily available measures were insufficient. This implies the need for better measures or better use of those measures. Regardless, the outcome of using custom measures is a lack of external confidence in the trust findings and difficulty in translating trust findings between studies.

While many new contenders have proposed improved measures that explicitly relate to trust models (Wojton et al.’s TOAST, 2020; Ullman and Malle’s MDMT, 2021) existing measures may be sufficient if used more appropriately. Yang et al. (2017) proposes that trust is better quantified by the “area under the trust curve” which captures changes in trust over time and in comparison with the automation’s reliability. Such a measure requires frequent sampling of trust, such as the single-item trust scale used by Yang et al. (2017) or the even simpler increase/same/decrease indicator deployed by Desai et al. (2013). These very brief scales enable trust to be captured dozens of times per experimental block and better capture dynamic variations in trust. While these measures provide greater granular sensitivity and less task interruption, they might be less reliable and valid than robust measures such as the Checklist for Trust (Jian et al., 2000). However, reduction in quality is not a guaranteed feature of short surveys; a single self-report item was shown to be just as accurate in measuring cognitive workload as much larger surveys (e.g., Monfort et al., 2018). Indeed, within custom measures of trust, we found single-item surveys to be overwhelmingly predominant. Single-item surveys can be acceptable if you consider that single items extracted from larger unidimensional scales are often equally able to predict relevant outcomes as compared to the larger scale in its entirety (Bergkvist and Rossiter, 2007, 2009; Monfort et al., 2018). However, single-item custom measures of trust are not necessarily derived from more well-established scales of trust. Moreover, the use of any single-item assessment remains somewhat controversial, as many researchers depend on measures of internal consistency to accurately assess scale validity. While custom scales may offer more practical advantages over longer scales, care must be taken to ensure consistency in the literature and that researchers are adequately capturing the construct of trust. It is thus recommended that in situations where trust needs to be assessed quickly and unobtrusively, the sample items should be extracted from larger scales and cited appropriately or that previously validated scales should be used. Future research should examine the degree to which single-item TiA scales are reliable and accurate compared to more thorough multi-item measures, in order to inform and support the use of the parsimonious measures.

Notably, the dimensionality of trust is a substantial point of debate on its own. Most self-report measures – especially single-item measures – utilize a unidimensional conception of trust. The scale may place trust and distrust on opposites ends of the scale as conceptualized by Jian et al. (2000) or conceptualize trust to no trust (Lee and Moray, 1992) However, there is contradictory evidence supporting trust and distrust as orthogonal constructs, with differing impacts on attitudes and behaviors (Lyons et al., 2011). Despite this evidence, unidimensional scales are standard in TiA research and form the theoretical basis of most trust models, including those discussed in detail in the introduction. While a complete analysis of trust’s dimensions is out of scope of the current work, it must be a consideration for anyone developing or evaluating scales.

In conclusion, the best scale to use is a matter of choice, but the context and trust construct must be considered. If a researcher was interested in evaluating the “trustworthiness” of a new technology, it may be the case that the well-established “Checklist for Trust” scale (Jian et al., 2000) or the less widely used “trust perception scale” (Schaefer, 2016) is perfectly adequate. If a researcher was interested in the dispositional trust of the operator (i.e., how a person *typically* trusts any technological agent), it is more appropriate to use the propensity to trust scale (Merritt et al., 2013). The takeaway message is that researchers should prioritize the aspect of trust that they are measuring – Trust attitude, Trustor’s Propensity, or the Factors of Perceived Trustworthiness – and the frequency of sampling needed and use those elements to determine the most appropriate measure.

### Behavioral Measures

Behavioral measures involve the observation and systematic recording of participants’ behavioral processes or tendencies. In the context of the current studies, behavioral measures capture interaction with the automated or autonomous system. However, that behavior includes a large variety of activities, encompassing those that may be intentional and active to unconsciously influenced and passive. To make sense of behavior over time, most of these measures are sorted into blocks or time periods. Nine different types of behavioral measures of TiA are reported here. These behavioral measures of trust are listed in Table 4 and defined in greater detail below, in alphabetical order. Representative sources are provided for each measure – these sources are commonly cited, but are not necessarily the origin of the behavioral TiA measure in question.

#### Combined Team Performance

If an automated teammate is consistently reliable and always accurate, then human teammate performance will be largely a product of the human’s trust, and therefore compliance, in the automation. Therefore, the combined team’s performance has been previously used as a proxy for behavioral trust in strictly controlled circumstances where the automation is reliable and performance is scored on the team’s correct or incorrect decision-making (e.g., de Visser et al., 2016). Performance is an indirect proxy, as it is subjected to external constraints and the competence of the human: An operator could theoretically avoid compliance with the automation while making correct decisions on their own. On an individual basis, or in uncontrolled scenarios, combined team performance may not accurately capture the human teammate’s trust. However, if all factors are controlled, then an increase in team performance suggests that the operator trusts and relies upon the unerring automation.

#### Compliance and Agreement Rate

Compliance is an active form of agreement, wherein the operator follows recommendations given by the system or positively responds to system alarms (Lee and Moray, 1994; Meyer, 2004). Compliance is typically calculated as the rate of agreement with system recommendations, warnings, or alarms over a given block of time or number of interactions. Compliance is sometimes distinguished from agreement by only counting instances when a human choice and an automated recommendation do not match, in situations when humans make an assessment prior to automation (Van Dongen and Van Maanen, 2013; de Visser et al., 2016).

#### Decision Time

Decision time refers to the length of time that it takes the participant to make a decision concerning the automation, often whether to comply with a recommendation. Faster decisions imply greater trust, while slower decisions imply the desire to consider or evaluate options before complying, thereby indicating distrust or a lack of trust (e.g., Yuksel et al., 2017). Some authors suggest that decision times may be a product of the mental evaluation method applied by the participant, with short and long decision times using different trust heuristics (see Alarcon and Ryan, 2018 for a processing model).

#### Delegation

Delegating a task to automation, when the task could be performed by a human operator, is a strong indication of trust in that automation. This behavior is characterized by the participant ceding control to the agent, rather than taking it away as in intervention. This measure is relatively novel but has been used to capture TiA in tasks where delegation to the automation is feasible (Xie et al., 2019).

#### Economic Trust Game: Stakes Invested

The Trust Game, invented by Berg et al. (1995), measures trust using economic decisions. The participant is paired with a teammate, given a quantity of money, and informed that sending money to their teammate may result in the sent value being tripled. The amount of money invested in the teammate is used as a proxy for trust. This measure is well-established even within the field of human-machine interaction (de Melo et al., 2013) and has recently been used to capture TiA (e.g., Mota et al., 2016; Wijnen et al., 2017). This task is often independent from the primary interaction with the automation and occurs following periods of interaction. As such, the trust expressed during this game represents the trust built up during the interaction. There are many other alternative trust games to Berg’s Economic Trust Game (1995) such as the ultimatum game (Güth et al., 1982), yet these have seen limited use in TiA research, despite a history of validation in interpersonal trust research. A discussion of alternative trust games is provided in Alarcon et al. (2021).

#### Intervention

Intervention is a behavioral opposite of Reliance, in which participants intervene and take over control from the teammate. This measure is identified as “takeover” by some authors. For example, recent work examined the degree to which drivers intervened in the operation of an automated parking system, which was associated with the degree of distrust in the system (Tenhundfeld et al., 2019, 2020). The act of intervention may be prompted by external events or a change in the participant’s internal state. The period of intervention may last for a single interaction or for an extended period of engagement. Lee and Moray, 1994 proposed that operators have a bias toward manual control, yet are loathe to switch between manual and automated control. Therefore, the act of intervening is indicative of a state of distrust that exceeds this hesitancy barrier.

#### Reliance

Reliance is a passive form of compliance, wherein the operator does not seek to correct or takeover the performance of the system (Muir, 1994; Meyer, 2004; Rice, 2009). Reliance is typically calculated as compliance to non-alerts – that is, the user not overriding automatic control in the absence of alert – over a given block of time or number of interactions. If the system is consistently reliable, then a strategy of reliance will increase team performance to 100%. Lee & See’s seminal study on trust and self-confidence with automated systems (1994) suggests that reliance is a behavioral product of operators’ trust in the automation exceeding their self-confidence at performing the task. Others have shown that high reliance can indicate a state of high or over-trust which is known as complacency (Parasuraman et al., 2008; Parasuraman and Manzey, 2010). In these situations, reliance is measured by the rate of detection of automation failures (Parasuraman et al., 1993). Yet, other authors have suggested that reliance may also be affected by external factors such as workload (Biros et al., 2004), where busy participants may default to reliance out of a deficit of mental resources as opposed to – or in addition to – a high level of trust.

#### Response Time

Response time refers to the length of time that it takes the participant to respond to an event, such as an alarm on a task that falls within the automation’s responsibility. The resulting reaction time between stimuli and response is an automatic process that largely bypasses conscious cognition. High trust results in longer reaction times because over-reliance on the automation causes the operator to be unprepared to respond, requiring greater mental effort and duration to switch tasks (Beller et al., 2013; Helldin et al., 2013; Körber et al., 2018). However, evidence for the effects of trust on response time has been mixed, and some authors recommend caution in using this measure (Chancey et al., 2015).

#### Verification

Verification is the act of confirming the accuracy of a teammate’s actions or recommendations and may precede compliance, reliance, or intervention. This act of double-checking the teammate’s advice or monitoring their performance is an indication of distrust (Ho et al., 2005) and may represent a relatively objective measure of trust (Moray and Inagaki, 2000; Bahner et al. 2008; Walliser et al., 2016). The incentive to verify before engaging in a trust behavior is often balanced by the cost of said verification: increased effort or lost time (Ezer et al., 2008). Researchers who deploy this measure of trust may include artificial penalties to simulate this cost (e.g., 30s time penalties for verification in Pak et al., 2012).

### Behavioral Measures Discussion

The categorization “behavioral trust” belies great depth and diversity. Trust behaviors may be passive (reliance), active (compliance), or engage in risk-taking in the relationship (posting stakes in the economic trust game). Trust behaviors even include active distrust, such as intervening or taking over from the automation. Trust is a predominant factor in both reliance decisions (e.g., Dzindolet et al., 2003) and compliance decisions (e.g., Rice, 2009), but these are different behaviors. Reliance is a passive behavior where the operator does not interfere with the automation’s actions, where compliance is an active behavior consisting of agreeing with and accepting the actions of the automation, whether that is a recommendation or an alert. The time between the alert and the operator’s response or decision – identified in our results as “decision time” – is similarly an active behavior that reflects trust. Faster responses imply greater trust, while slower responses imply the desire to consider or evaluate before complying, thereby indicating distrust or a lack of trust.

Operators may also engage in active trust behaviors, taking risks such as investing monetary stakes in their partner in the economic trust game or delegating tasks to the automation. A wide variety of trust games other than the economic trust game is available to researchers – while many have not yet been validated in a human-automation trust scenario, they would expand the active trust measures available to researchers. See Alarcon et al. (2021) for a discussion of these trust games. Whether using trust game or delegation action, these behaviors indicate a strong trust attitude, as each behavior represents a willingness to take on a great degree of risk and vulnerability in the hope that the teammate will have the ability to perform and the benevolence and integrity to reciprocate.

Using trust-related behaviors to capture trust is risky: Although trust affects behavior, it is not the sole influence. Complying or relying on automation may be a consequence of high workload (Biros et al., 2004) rather than a strong internal attitude of trust. Similarly, these behaviors may be influenced by risk: Users become more self-reliant when risk is high (Ezer et al., 2008). The behavioral trust measure of performance is similarly affected. While this measure captures the net performance of the automation and the human operator working together, over-reliance or compliance due to high workload may lead to the false conclusion of high trust. Conversely, the behavioral measures of verification and intervention are measures of distrust or lack of trust, as the operator should only interfere with the automation when their trust is lower than their self-confidence in manually performing the task (e.g., Lee and Moray, 1994). As with the prior behavioral measures, workload is an influencing factor, though in this instance intervention is more feasible when workload is low, not high. Indeed, there is even some evidence that verification may become the norm when the operator is under-stimulated (e.g., Walliser et al., 2016).

Overall, these behavioral trust measures capture Risk-Taking in Relationship, as identified within Mayer’s trust model (1995). Strictly speaking, they are not measures of the Trust construct, as the influence of Perceived Risk and context help differentiate Risk-Taking in Relationship from the Trust construct. Regardless, as an indirect product of the internal trust attitude, behavioral measures are considered trust measures in common parlance. This discussion continues with a review of their pros and cons, as well as recommendations for their use.

#### Critiques, Considerations, and Recommendations for the Use of Behavioral Measures

Regardless of whether trust behaviors are active or passive, experimenters must be aware of the advantages and disadvantages of such measures. Perhaps one of the greatest benefits of behavioral measures is the ability to integrate a robust and reliable measure of trust into experiments with minimal disruption: Compliance, reliance, response times, and rate of interventions are often fundamental features of tasks performed with automation or autonomous systems. The addition of these measures requires only a mechanism to record them for the sake of empirical measurement. Other behavioral measures, such as investment in the economic trust game or delegating to automation require intentionally designing the experiment to accommodate the behavior, yet offer a greater signal of trust state in exchange. Each behavior represents a willingness to take on a great degree of risk and vulnerability in the hope that the teammate will have the ability to perform and the benevolence and integrity to reciprocate. The act of delegation, for instance, requires the user to trust the automation more than their own ability to perform the task – this behavior is more active than other trust behaviors such as reliance, which may be completely passive.

Most behavioral measures are capable of sampling trust at a much higher rate than many self-report measures, as they capture every trust behavior that occurs during the task. As such, behavioral measures offer the opportunity to more readily capture the “area under the trust curve” as defined by Yang et al. (2017). However, utilizing this high sampling rate and capturing the dynamic variation of trust requires analysis methods that are less commonly used and debatably less accessible, such as bi-modal regression or other non-parametric analyses. Behavioral measures are often instead analyzed as mean rates of behavior over time periods, which reduces their time sensitivity in favor of easier parametric analysis – four instances of compliance and one instance of non-compliance can thereby be transformed into a “trust rate” of 0.8 across the given five-decision-point block. This shortcut of converting frequency counts into proportions may introduce error into the statistical outcomes and represents only one of many methods by which behavioral trust measures may be abused in analysis. Behavioral measures are also limited by the presence of extraneous variables such as workload and risk level, which are a threat to external validity. To a degree, this is manageable *via* traditional experimental controls, but many studies do not verify the validity of their behavioral trust measures beyond these controls. While these measures have been validated and are confirmed to be capable of measuring trust, that is no guarantee that they will capture trust in every given study. Therefore, researchers should seek to capture and control known extraneous variables and confirm that the behavioral trust measure of choice correlates with other trust measures. Using validated self-report measures of trust may be one way to establish convergent validity and offers the added benefit of providing insight into how perceptions of the automation’s trustworthiness influence these trust behaviors. These practices are relatively uncommon in the current state of research.

To further guarantee that trust is actually being measured and manipulated, researchers should ensure that the situation tested is one that produces a certain degree of uncertainty and vulnerability. Prior TiA studies has successfully introduced these characteristics, whether manipulating risk by threating virtual convoys with explosive attacks (Lyons and Stokes, 2012) or simulating escaping a burning building with a robot aid (Robinette et al., 2016). In conjunction with standard experimental controls, these efforts can limit the effect of extraneous variables and amplify the influence of trust.

As mentioned earlier, there are several advantages offered by behavioral measures of trust, including the unobtrusive nature of acquiring relevant data, the robust nature of the response, and the ability to eliminate the bias associated with the use of self-report. However, behavioral indices of trust highlight why trust is difficult to measure directly. For example, it is highly likely that a user can distrust a system but still exhibit reliance behaviors, such as when inadequately trained or when workload is high. Trying to understand trust through behavior is a premier example of the inverse problem in psychology: trying to infer from a set of observed behaviors the causal factors that created those behaviors. Therefore, some authors have attempted to measure trust as an intention as opposed to a behavior *per se* (e.g., Lyons and Stokes, 2012). The issue is further compounded by the fact that trust behavior manifests differently depending on the “level of automation.” For example, with an automated decision aid, you can decide to comply with the recommendation or not. It is far more complex of a decision in the case of an autonomous system. In this context, when there is distrust, the operator must decide whether they will assume complete control over the system. It could very well be the case that there are multiple decision pathways involved in the latter and not the former, thereby rendering compliance and takeover behavior incomparable. Thus, due to the opaque nature of behavioral measures of trust, the authors recommend that behavioral measures – in whatever form they take – be coupled with more direct measurements of trust such as self-report. This may help to parse out the variability in behavior that is likely due to “trust” and not some other factor (e.g., workload).

### Physiological Measures

Physiological measures capture biological responses ranging from muscle movements to hormonal levels to neural activation. Eye movements are also included in this category. As these measures capture the results of complex cognitive processes, they must be used in combination with context of when trust is relevant and with hypotheses concerning how these measures will change to reflect trust states. Otherwise, researchers may capture results that are orthogonal to trust or cannot be related back to trust states. Four different types of physiological measures of TiA are reported here. These physiological measures of trust are listed in Table 5 and defined in greater detail below, in alphabetical order. Representative sources are provided for each measure – these sources are commonly cited and archetypal uses of each method.

#### Electrodermal Activity

Electrodermal activity (EDA) – also known as galvanic skin response – is the measurement of sweat gland activation *via* skin conductivity (see Sharma et al., 2016). These methods use skin contact electrodes to detect increases or decreases in ionic activity triggered by sweat, where sweat increases due to strong states of emotional arousal, including positive emotional stimuli, stress, anxiety, and high cognitive workload (Nikula, 1991; Jacobs et al., 1994). Measurement in the hand region is particularly sensitive to stress and engagement (Mower et al., 2007). As such, EDA has been used as a proxy for TiA. EDA corresponds with increased engagement with robots (Bethel et al., 2007), and the levels of EDA have been shown to be affected by level of trust (Khawaji et al., 2015), which provides support for their result use as a trust measure (e.g., Akash et al., 2018). Placing electrodes on the hands does limit the interactions that can be performed with this method, but minor task modifications or using the participant’s non-dominant hand reduce those limitations. Because EDA response requires emotional arousal, difficult or stressful tasks that may be mitigated or exacerbated by the automation’s involvement are recommended to take full advantage of this method.

#### Eye Gaze Tracking

Eye movements, known as saccades, are a physical expression of attention (Zhao et al., 2012) and therefore have been used as a measure of whether participants are monitoring the automation. Monitoring of an automated system has an inverse relationship with trust (Hergeth et al., 2016) and a positive relationship with distrust (Tenhundfeld et al., 2019) – Hergeth suggests that tracking gaze behavior provides a more direct measure of automation trust than many other behavioral measures.

The primary trust measures derived from eye gaze tracking – also known simply as eye tracking – are the frequency and duration of eye fixations in given areas of interest: If the participant looks at the visual area containing the automation’s process, task, or outcomes more often, they are perceived to have less trust in the automation (Hergeth et al., 2016). As a caveat, participant’s glances into the area of interest are only relevant when trust in the automation is relevant. This measure is functionally a physiological expression of the behavioral measure of monitoring or verification behavior. Other measures have utilized the location and pattern of eye movements (saccades) to indicate trust (e.g., Gold et al., 2015), but these results have been inconsistent.

Eye tracking may be inserted into tasks which have distinct visual areas containing the automation or automation’s task. As such, this measure is well-suited to tasks performed by autonomous systems which have complete control over a given procedure, such as a factory process or self-driving vehicle, or where automation is responsible for a procedure on a discrete portion of the interface or environment. In both instances, glances into that area during periods when trust is relevant likely represents monitoring triggered by low trust or distrust, rather than the operator performing their primary task. The resulting data are recorded on a timescale of milliseconds and are often analyzed as a percentage during time blocks or after critical events. The quality and accuracy of said data often have an inverse relationship with the intrusiveness of the eye tracking hardware, but rapid improvements in eye tracking technology are replacing chin mounts, long calibration times, and heavy head gear with lightweight tools. Current generation eye trackers are minimally intrusive and can enable eye tracking in a real-world 3-D environment rather than a 2-D screen, expanding the possible applications of this measure.

#### Heart Rate Change and Variability

Heart rate may be used as a physiological expression of emotional or mental arousal, especially workload and stress (Payne and Rick, 1986). Given that an operator’s workload and stress should decrease if they are working alongside a teammate they can trust, variations of heart rate have been used as a measure of trust. Heart rate change was used successfully in a composite measure of TiA by Waytz et al. (2014) under the hypothesis that humans who trust their self-driving vehicle will experience attenuated heart rate increases during a vehicular accident when the self-driving vehicle is in control.

Heart rate variability is a more complex measure focusing on the variation in time between each heartbeat. This variability is controlled by the autonomic nervous system and in turn the sympathetic and the parasympathetic nervous system. These systems are more popularly known as the fight-or-flight and relaxation responses and respond to stress and wellbeing. Stressed individuals are likely to have little variation between heart beats due to sympathetic activation, while relaxed individuals will have high variation as their heart beats quickly adapt to temporary physiological or psychological activation. Therefore, heart rate variability (HRV) has been used to extrapolate high levels of workload (Aasman et al., 1987; Wilson, 1992), and in combination with other physiological metrics such as EDA, HRV has been shown to capture trust in other humans (Montague et al., 2014). However, attempts at using this measure to capture TiA have found limited success (e.g., Petersen et al., 2019). While heart rate-derived measures of trust would provide a relatively unobtrusive measure for high workload or high-stress tasks performed alongside automation, empirical support for this measure is mixed. When these metrics have been utilized to capture trust, they have either focused on trust between humans or with heart rate as part of a larger composite physiological-behavioral measure.

#### Neural Measures

The attitudes and behaviors related to trust have cognitive components, which can theoretically be captured and used as a measure of trust. However, the application of neural measures to trust is relatively new and somewhat exploratory, especially when it comes to TiA. The methods deployed include electroencephalogram (EEG), functional magnetic resonance imaging (fMRI), and functional near-infrared spectroscopy (fNIRS). While the methods differ, all capture the same thing: the location and degree of brain activity that is associated with TiA.

The EEG is a tool used to locate and measure electrical activity in the brain using electrodes resting on the scalp (Jackson and Bolger, 2014). The fMRI, using a magnetic field, and fNIRS, using near-infrared light, both capture brain activity by detecting oxygenation and deoxygenation of the blood, as increased neural activity demands more oxygen and thereby increased blood flow (Sylvester et al., 2003; Tsunashima and Yanagisawa, 2009). The fMRI uses a magnetic field for measurement, while the fNIRs uses near-infrared light. All three methods have different trade-offs in terms of regional accuracy, temporal specificity, and signal type and are often paired to establish convergent validity. These methods capture activity in brain regions and networks for regions that correlate with trust and are therefore used as a proxy for TiA. EEG signals have indicated surprise and violation of expectations while monitoring imperfect automation or algorithms (Akash et al., 2018; de Visser et al., 2018; Somon et al., 2019) and can differentiate between human-like agents (Dong et al., 2015; Wang et al., 2018; Jung et al., 2019). fMRI signals have differentiated brain regions and networks associated with observation of errors with machines (Desmet et al., 2014), the tendency to comply with automation (Goodyear et al., 2016, 2017), and differences between human-human trust and human-machine trust (Riedl et al., 2011, 2014). fNIRS has been used to characterize suspicion and trust (Hirshfield et al., 2014). Neural measures of TiA have not yet been widely adopted, likely due to their exploratory nature and the mandate to use specialized hardware and training. However, these methods enable the real-time collection of high-quality data that likely captures trust attitudes and cognitive states that relates to trusting behaviors. The experiments cited above have deployed these measures in the types of interactive tasks that are common in human factors experiments and have required little adaptation or interruption of the task other than the set-up and deployment of the hardware. We anticipate that this method will become more widespread as these measures are formalized, validated, and become more accessible to non-neuroscientists.

### Physiological Measures Discussion

There are currently two broad types of physiological measures used to capture TiA: neural measures and several types of physiological reactions. Neural signals encompass activity in the brain captured by a variety of measures, such as fMRI or EEG, while physiological reactions encompass a wider range of measures, ranging from eye gaze tracking to heart rate change and EDA. Physiological measures provide a glimpse into the neural and physiological underpinnings and correlates of trust. As such, they may therefore provide direct measurement of the internal attitude of Trust and the mental processes underlying risk-taking, using the constructs defined in Mayer’s trust model (1995).

#### Critiques, Considerations, and Recommendations for the Use of Physiological Measures

Physiological measures have many potential advantages for trust researchers. They collectively represent an opportunity to measure trust in real-time, with potentially greater sensitivity than self-report or behavioral measures. In contrast to self-report measures, they are less invasive in terms of disrupting the flow of interaction. In contrast to the artificial task scenarios used to enable behavioral measures, physiological measures facilitate a different and potentially broader set of tasks used to manipulate trust.

However, physiological measures have a unique set of disadvantages that lead many practitioners to eschew their use. Every one of these measures requires specialized hardware and training to use said hardware and organize the resulting data. Furthermore, outcomes must be contextualized during periods where trust is active and relevant, and *a priori* hypotheses must be established concerning how these measures will change during trust episodes. Without this groundwork, researchers may be unable to differentiate between variation caused by trust and that triggered by exogenous variables. Even worse, the latter may be incorrectly identified as trust in the analysis. In short, physiological measures require a large amount of expertise and planning to apply correctly.

Despite these difficulties, the use of physiological trust measures is perhaps one of the fastest growing areas of TiA measurement, in part due to the plethora of pre-existing physiological measures that can be drawn from human-human trust measurement and the growing field of neuroergonomics that embraces this methodology (Parasuraman, 2003, 2011; Gramann et al., 2017). Neural measures allow researchers to leverage the vast body of neuroscientific knowledge that has accumulated with respect to trust in humans (de Visser and Krueger, 2012; Bellucci et al., 2017; Krueger and Meyer-Lindenberg, 2019). Neuroscientific research using EEG and fMRI has previously established brain regions that contribute to the trust process (Krueger et al., 2007) and signals that indicate intention to trust (Delgado et al., 2005; King-Casas et al., 2005) while using tasks that would be easily transferred from human to automated partners. More specifically, researchers have proposed that neuroscientific measures can be informative for supervision and monitoring of errors (Fedota and Parasuraman, 2010; Berberian et al., 2017), understanding the antecedent decision prior to trust interaction behaviors (Drnec et al., 2016), assessing the effectiveness of human-automation etiquette (de Visser and Parasuraman, 2010; de Visser and Parasuraman, 2011), as an input for brain-computer interfaces (Strait and Scheutz, 2014), evaluating the viability of social robots (Wiese et al., 2017; Henschel et al., 2020), and understanding differences between humans and machines that have human-like properties (Krach et al., 2008; Kircher et al., 2009; Saygin et al., 2012; Wang and Quadflieg, 2015; Hoenen et al., 2016; Özdem et al., 2017; Rosenthal-von der Pütten et al., 2019; Moreau et al., 2020). While we should not minimize the effort required to formalize and validate these measures of trust, adapting them to automation avoids re-inventing trust measures and quickly expanding the neural measures available to TiA researchers.

Some researchers are attempting to expand physiological measures even further by exploring the role of hormones – particularly oxytocin – as a potential route of trust influence and measurement. Oxytocin has previously been administered to enhance trust in humans (Kosfeld et al., 2005), and oxytocin measurement can indicate the degree to which people trust or bond with canines (Nagasawa et al., 2015). Such approaches have recently been applied to TiA or social robots. One study explored the effects of administering oxytocin on trusting human-like agents such as a computer, an avatar, or a simulated human that were either perfectly or 50% reliable. Results for this study showed that administration of oxytocin only influenced behavior toward a perfectly reliable avatar and not any of the other agents (de Visser et al., 2017). Although there has been recent criticism of the oxytocin methodological and experimental approach due to reproducibility difficulties and the exploratory nature of this work (Christensen et al., 2014; Nave et al., 2015; Mierop et al., 2020), a validated hormone-based measure would present a third broad type of physiological trust measurement. Further research into smell and chemical signaling that influences trust in robots may extend this measurement further (e.g., van Nieuwenburg et al., 2019). It is difficult to make a single recommendation for the use of physiological measures, given the diversity and relative novelty of measures contained within this category. Therefore, we have reviewed the potential use of all four measures.

Eye tracking measures are thus far the most common physiological measure of TiA. This method is relatively flexible and can be integrated into many existing tasks. Automation-assisted driving is a common application (Hergeth et al., 2016), though any task that involves participants’ TiA – and enables the participant to visually monitor that automation during the task – is eligible for eye gaze tracking. Recent developments in headset-free eye gaze tracking make this measure even less disruptive to the original task, which makes it a prime candidate for measuring trust in real-world or non-artificial scenarios. The real-time nature of the measurement makes this method a valuable supplement to behavioral measures of trust. Eye tracking is also a much more established measure compared to other physiological measures, with researchers able to reference previously published works as a guide.

Electrodermal activity and HRV and change are relatively novel methods for trust measurement. While they promise a relatively real-time measure of trust, their use as a trust measure has been infrequent and the results unclear. These are sympathetic responses in reaction to stress, where stress is hypothesized to decrease when collaborating with a trusted partner. Theoretically, these measures would be best applied in high-stress situations where high trust would indeed reduce stress, providing a semi-real-time correlation to the internal trust attitude. However, we caution that these measures remain exploratory and are unlikely to ever provide more than a moderate correlate of trust. They should be combined with other proven measures of trust and used with caution until further empirical validation has been performed.

Neural measures purport to capture variations in trust attitude or cognitive processes that relate to trust attitude. If successful, a neurological measure of trust would be the most sensitive, real-time capture possible for this latent variable. Sampling neural activity can pick up changes very shortly after they occur, without the decision-making delays of behaviors. They can capture implicit attitudes in contrast to the biases that influence self-report. Finally, neural measures are minimally task-invasive and require only that the participant can complete the procedure while burdened or confined by the requisite EEG, fMRI, or fNIRS hardware. While the hardware is constraining, the techniques referenced in this review theoretically enable trust to be captured during most types of trust-reliant tasks. Regardless, we recommend utilizing these methods with caution and a thorough understanding of the underlying literature. Neural measures of trust have a relatively long history of use within interpersonal research, but their application to automation is relatively novel, and often experimental.

These four physiological measures of TiA each have their own potential usage and have found varying degrees of success in capturing TiA using the same types of tasks that are widely used throughout the fields of human factors and human-machine interaction. Overall, we recommend using these measures strategically to support behavioral and self-report measures of trust. Eye tracking measures are highly recommended for tasks that trigger behavioral expressions of trust, due to their real-time data capture and ability to measure trust in ecologically valid scenarios. Due to the need for further validation, we recommend neural measures primarily for experimenters who are exploring the mechanisms of trust itself or are otherwise limited by task type. These measures should likely be avoided for routine trust experiments until further validation has been performed, given the level of effort and uncertainty required. Finally, we recommend against relying on EDA, HRV, or their derivative measures as primary trust measures until more validation has been performed. These methods have theoretical potential, but limited success in practice.

Overall, we see substantial promise in physiological measures of TiA. While this research trajectory is in its relative infancy, these measures may be capable of capturing trust with minimal task alternation or interruption and in doing so may provide a real-time measure of trust that captures the moment-to-moment variation that is intrinsic to the definition of trust as a process (e.g., Mayer et al., 1995).

## Discussion

This review covers the methods used to capture and analyze TiA, accounting for self-report, behavioral, and physiological measures. To better understand how these measures are applied and the trade-offs inherent in each measure, we discuss these categories of measurement in greater detail. Furthermore, we provide recommendations regarding the application of trust measurements.

### General Measurement Recommendations

With 30 different measures of TiA identified in this review, researchers have a plethora of methods available, arguably sufficient to capture trust in nearly any task and situation. Despite this, trust is too frequently poorly measured and new measurement methods are being constantly created, often without validation or properly referencing prior works.

The discrepancy between the possible methods and the limited set of methods actually deployed suggests the simple need for awareness, as alluded in the introduction. Researchers should have a reference list of all types of measurement that are possible, facilitating their choice of the most appropriate method rather than the most familiar. This is especially salient given that researchers seem to be creating their own new measures rather than using pre-validated measures – prior work found that 31% of reviewed measures used a custom Likert or slider scale to capture TiA, and most of these measures contained a single item (Kohn et al., 2020). While we do not claim insight into the cognition of other researchers, given that these custom single-item trust scales are similarly worded to established parsimonious scales (e.g., Lee and Moray, 1992, 1994; Muir and Moray, 1996), it is likely that the perceived need to create entirely new scales is due to lack of a coherent reference guide rather than preference. This reference work would theoretically reduce some of this effort and redundancy.

Regardless, the issue of insufficient measurement remains. Based on our review and the process of contextualizing measure within models, we recommend the following actions to improve the deployment and discussion of trust measurement:

1. Use measures that have face validity
2. Contextualize experiments and measures within trust models
3. Understand what the chosen measure captures before using it
4. Understand that different trust measures may capture different facets of trust
5. Establish convergent validity with multiple measures

This review provides the knowledge base required to achieve all five recommendations. Possible validated measures are listed for practitioners, each associated with specific model components that explain the facet of trust that they capture. While the original measure might have described the outcome as “trust,” our list defines that same outcome as specific components such as Factors of Perceived Trustworthiness (Mayer et al., 1995) or Risk-Taking in Relationship (Mayer et al., 1995). These concepts are indeed related to trust but grouping them as that singular construct belies the ground truth that these are different components influenced by different factors and will have different outcomes. Ignoring this complexity may lead researchers to view the outcomes of these different measures as contradictory and poor quality, when in fact the inconsistency between the outcomes of Jian’s Checklist for Trust (2000) and reliance exists because they capture different elements of the trust process. Future meta-analyses could use this taxonomy to understand and assess to what degree researchers have studied the different factors in the trust process, leading to a better understanding of the current state of TiA research.

Similarly, researchers and practitioners can now easily compare and contrast trust measures and deploy measures of trust that are either convergent or cover different components of trust. While the recommendation for convergent validity has not been thoroughly covered in this work, capturing multiple dimensions provides a more robust set of measurements that is more resilient against the effects of confounding variables and can explain participant behaviors in greater detail. A combination of behavioral and self-report measures, for instance, may serve to validate each other and provide greater evidence of the automation’s behavior on trust. Examining the current body of work, a great number of researchers deploy a single measure of trust – often behavioral – and declare that it is capturing trust without sufficient evidence or validation.

While we provide these recommendations and an educational tool with the goal of improving the state of trust measurement, there remain three systematic needs which beg for solutions: the need to understand whether single-item self-report trust measures are effective compared to longer surveys, the need to determine whether these measures work equally well for different levels of automation and robotic embodiment or social presence, and the need for a better categorization or model in which trust measurement may be discussed.

First, we believe that researchers may be seeking lightweight and unobtrusive trust measurements, based on the aforementioned frequency of custom self-report trust measures, and the fact that most of those measures feature only a single query (Kohn et al., 2020). Fortunately, this review work identifies several minimalistic self-report methods such as Lee and Moray’s two-item trust and self-confidence survey (1994) or Desai’s dynamic trust reporting (2012). These measures may be sufficient for many researchers’ needs but should be empirically compared to larger checklists such as the Checklist for Trust (Jian et al., 2000) or the trust perception scale (Schaefer, 2016). With 12 and 14 items, respectively, these longer surveys advertise thoroughness, reliability, and accuracy in trust measurement. While they likely have advantages over brief one- or two-item scales, the relative effectiveness of these options has not been thoroughly compared. For example, a single-item measure evaluated workload just as well as the six-item NASA-TLX (Monfort et al., 2018). This study also found that repeated administration of the six-item scale increased workload itself by 18%. This demonstrates a significant cost for administering multi-item measures repeatedly in an experiment. In the meantime, however, researchers should consider utilizing alternative lightweight trust measures, such as behavioral or physiological measures.

Second, there is a substantial need to determine whether the trust measures reviewed here are effective across different levels of automation, physical or virtual embodiment, and mind perception. These factors have been shown to influence trust (e.g., Walliser, 2011; de Visser et al., 2016, 2017; Pak et al., 2012) and may therefore influence the effectiveness of the trust measures themselves. This becomes an issue when trust measures intended for humans or robots are deployed for TiA or used to compare some combination of the above. We suggest that these measures may not be equally applicable to all agents: For example, all else being equal, automation can lack robots’ anthropomorphic characteristics and humans’ capacity for integrity. Some measures take account of these differences, such as Malle & Ullman’s MDMT measure (2021) which enables participants to opt out of answering if the term is not applicable to the current agent. Such measures are few and far between. Effort should be undertaken to understand whether popular trust questionnaires are equally applicable and universal or whether the results are dependent on the nature of the agent in question, independent from trust. Some initial efforts have been made to explore the efficacy of different trust questionnaires in different scenarios (e.g., Chita-Tegmark et al., 2021) – sufficient evaluation of available measures could enable the creation of an objectively based flowchart for selecting trust measures based on agent and task criteria.

Finally, while we recommend that all measures should be created and discussed in the context of trust measures, we also recognize that existing models may not be ideal for this discussion. Some of the most popular trust models are not empirically supported, and many do not easily map to existing measures of trust. Therefore, we propose that a new model or categorization may be required. However, the path toward this model is unclear. It would be difficult to create a model that encompasses all facets of TiA, given its complexity and context-specific nature. We do suggest one possible route: a model based on the measurable components of trust. The existing models and measures reviewed in this work are primarily centered around three components: judgments of trustworthiness; trust attitudes; and trusting behavior. Such components could be defined in detail and form the basis of a future parsimonious framework to discuss and categorize trust measures, minimizing the jargon and elaborate definitions inherent in many trust models. Ultimately, such a model may better integrate the many ongoing research efforts to investigate TiA and more efficiently advance our scientific understanding of this important topic.

## Summary and Conclusions

Our review of existing methods of measuring TiA provides an educational tool for practitioners who are new to the field and for established researchers who would like to utilize the optimal method for their task and hypotheses, while understanding the limitations of different methods. We also suggest the need for improved trust measurement beyond simply selecting better methods – trust-oriented research should sample trust more frequently to capture the “area under the trust curve” (Yang et al., 2017) and use multiple measures for convergent validity. Furthermore, trust researchers should *a priori* identify and attempt to capture specific components of trust, such as those established in Mayer’s process-oriented trust model (1995). Identifying the specific component that is being captured will help researchers better predict and explain their data, as well as its place in a complex trust system. However, we acknowledge that the tendency to claim that overall “trust” is being captured, rather than a specific component of trust, may be due to an intrinsic weakness in many trust measures: The measures themselves claim to capture a monolithic construct of trust. Few explicitly or implicitly relate to specific components of any trust model. The onus of responsibility may lie on the creators of trust measures to relate them to existing models, or perhaps for a better model that coherently maps to trust measures. A model where each component is clearly defined and measurable could vastly improve the analysis and discussion of TiA. In the meantime, we hope that this review – and the discussion within – will provide the tools for researchers to improve their own measurement of TiA.

## Author Contributions

SK and TS conceptualized this work with assistance from EV. SK conducted the search, inclusion process, and review of relevant works, with guidance from TS and EV. All authors assisted with conceptualizing the organization of measures, relating the relevant theories and models, and directing the discussion. SK drafted the manuscript with contributions from all other authors. All authors contributed to the article and approved the submitted version.

## Conflict of Interest

The authors declare that the research was conducted in the absence of any commercial or financial relationships that could be construed as a potential conflict of interest.

## Publisher’s Note

All claims expressed in this article are solely those of the authors and do not necessarily represent those of their affiliated organizations, or those of the publisher, the editors and the reviewers. Any product that may be evaluated in this article, or claim that may be made by its manufacturer, is not guaranteed or endorsed by the publisher.
